# Epidemiological, clinical characteristics and drug resistance situation of culture-confirmed children TBM in southwest of China: a 6-year retrospective study

**DOI:** 10.1186/s12879-020-05041-3

**Published:** 2020-05-01

**Authors:** Dong-Mei Wang, Qing-Feng Li, Ma Zhu, Gui-Hui Wu, Xi Li, Yuan-Hong Xu, Jing Zhong, Jia Luo, Ying-Jie Li, Bin-Wu Ying, Chuan-Min Tao

**Affiliations:** 1 Department of Laboratory Medicine, West China Hospital, Sichuan University, Chengdu, 610041 China; 2 Department of Clinical Laboratory, Public Health Clinical Center of Chengdu, 377 Jingming Road, Jinjiang District, Chengdu, 610061 Sichuan China

**Keywords:** Epidemiology, Clinical features, Drug resistance, Tuberculosis, Meningeal, Child

## Abstract

**Background:**

Sichuan is a province located in southwestern China, which have a higher incidence of tuberculosis (TB). This study aimed to analyze the epidemiological and clinical characteristics, as well as drug resistance in culture-confirmed children with *Tuberculosis meningitis* (TBM) in Southwest of China.

**Methods:**

We performed a retrospective study on children (< 14 years old) with cerebrospinal fluid (CSF) culture-confirmed TBM between January 2013 and December 2018 at Public Health Clinical Center of Chengdu (PHCCC). *Mycobacterium tuberculosis* (MTB) drug sensitivity testing (DST) was performed using the MicroDST™ method. The age, gender, family history of tuberculosis, status of Bacillus Calmette–Guérin (BCG) vaccination, residential areas information, clinical, laboratory, and radiological features were recorded. Data were analyzed using SPSS Statistics Client 25.0, and the change in drug resistance rate was examined using the Cruskal-Wallis test.

**Results:**

Among 319 patients clinically diagnosed with TBM, 42 (13.2%) were Mycobacterial culture positive. Their median age was nine years, and the distribution was equal among female and male patients. Among 42 patients who were enrolled in the study, 1/42 (2.38%) passed away. Children with TBM were concentrated in the minority areas of western Sichuan, where 34/42 (81.0%) patients with TBM belonged to ethnic minorities, and only 2/42 (4.76%) received BCG vaccination in the past. Chest X-rays changes were observed in all patients. Fever and headache were the most common presenting symptom. Thirty-five (83.3%) patients suffered from neck stiffness, and 30/42 (71.4%) had high CSF pressure. DST results showed that the resistance rate was high; resistance to any anti-tuberculosis drug (ATD) was observed in 13 (31.0%) patient isolates, while multidrug-resistant TB (MDR-TB) and extensively drug-resistant TB (XDR-TB) were found in 2 (4.8%) and 1 (2.4%) patients, respectively.

**Conclusions:**

TBM among children in Southwest China was mainly concentrated in the minority areas of western Sichuan and more than 95% of patients did not receive BCG vaccination at birth. The most common symptoms were fever, headache, and neck stiffness and all patients had positive chest X-ray findings. In addition, high rates of drug resistance were found.

## Background

According to the latest statistics, there were estimated 1.2 million Tuberculosis (TB) deaths among HIV-negative people in 2018. Among those, 14% were in children younger than 15 years [1]. TBM with the case fatality rate of 30% is among the most severe types of extra-pulmonary tuberculosis. In our previous studies, we have found that TBM accounts for about 8.1% of all culture-confirmed TB cases [2]. Meanwhile, children’s tuberculosis morbidity and mortality have the highest rates among the infectious diseases in China, with severe tuberculosis, TBM and multiple drug-resistant TB being on the rise.

Sichuan is a Chinese province located in the southwest of China. This area is also known as Minority Enclaves. Because of the remote geographical position and control paucity of local medical services, this area has very high incidence of TB. Consequently, identifying the epidemiological and clinical characteristics, drug resistance and the geographical distribution of children with TBM can provide a scientific basis for the prevention, control, diagnosis, and treatment of the disease.

## Methods

### Ethics approval and informed consent

This study was approved by the Ethics Committee Public Health Clinical Center of Chengdu, Sichuan, China [AF/SC-08/01.0], project approval No.[2017Y]025. As this was a retrospective study and all patient information used in this study were routinely collected through mandatory notification system, the ethics committee waived the requirement for informed consent.

### Study population

Sichuan province lies in the southwest of China and is one of China’s largest provinces. Our study was carried out at the PHCCC. This institution is the authorized hospital for treating TB in Southwest China (population around 89 million).

This retrospective study enrolled consecutive CSF culture-positive *Mycobacterium tuberculosis* cases that were confirmed and treated at the PHCCC between January 2013 and December 2018. TBM was diagnosed based on the Chinese Pulmonary Tuberculosis Diagnostic Criteria (WS 288–2017), the Chinese’ TB volume of clinical diagnosis and treatment guidelines’ (Chinese Medical Association, 2005), and the updated World Health Organization (WHO) guidelines [3]. A total of 319 potential children patients with TBM were recruited, 42 (13.2%) were Mycobacterial culture positive. The medical records of all 42 patients with culture-confirmed TBM were reviewed; data was collected on demographics, clinical, radiological and laboratory findings at presentation and outcome, including ATD toxicity. ATD induced hepatotoxicity was defined as serum alanine aminotransferase (ALT) ≥ 3 times the upper limit of normal (ULN). BCG vaccination status was determined by self-report and forearm scar examination.

### Bacterial strains culture, identification and drug sensitivity

BACTEC MGIT 960 system (Becton Dickinson & Co., NJ, USA) was used for Mycobacteria culturing. Clinical sterile CSF samples were collected, low volume CSF samples (< 0.5 ml) were directly inoculated into BACTEC MGIT 960 culture tubes. Where the CSF volume was > 0.5 ml, the upper membrane and lower 0.5 ml, including precipitates, were inoculated into the BACTEC MGIT 960 culture tube. WHO recommended neutralization, and the centrifugation method was used for the purulent or bloody CSF specimens. DST of the culture-positive MTB isolates was performed using MicroDST™ (Yinke AUTOBIO diagnostics Co., Ltd., Zhuhai, China) following the manufacturer’s instructions. The following first and second-line drugs were applied: isoniazid (INH, 0.4 μg/mL and 1.6 μg/mL), rifampicin (RIF, 2.0 μg/mL and 8.0 μg/mL), streptomycin (STR, 2.0 μg/mL and 8.0 μg/mL) and ethambutol (EMB, 5.0 μg/mL and 20.0 μg/mL); fluoroquinolone drugs ofloxacin (OFX, 1.5 μg/mL and 2.0 μg/mL), levofloxacin (LFX, 2.0 μg/mL and 8.0 μg/mL), and moxifloxacin (MFX, 0.5 μg/mL and 2.0 μg/mL). In addition, oral bacteriostatic second-line ATDs (prothionamide (PTO, 10.0 μg/mL and 40.0 μg/mL), rifabutin (RFB, 0.75 μg/mL and 3 μg/mL); and the second line parenteral agents (injectable ATDs) amikacin (AMK, 1.0 μg/mL and 4 μg/mL) and capreomycin (CM, 2.5 μg/mL and 10 μg/mL) were used. The control strain H37Rv was monitored. P-nitrobenzoic acid (PNB) and thiophene-2-carboxylic acid hydrazide (TCH) were first used for MTB identification, while TB-DNA (CapitalBio Corporation) was also used for further identification of species/complex levels.

### Laboratory quality control

External quality assessment (EQA) was conducted at the National Tuberculosis Reference Laboratory of the Chinese Center for Disease Control and Prevention. EQA included smear, culture, and DST. Blinded retesting of a random selection of ≈10% of isolates from the study laboratory was conducted in a superior laboratory.

### Statistical analysis

Data were analyzed using SPSS Statistics Client 19.0 (SPSS Inc., IL, USA). The measurement data of normal distribution were expressed as median or mean ± standard deviation, and categorical variables were expressed as the number and percentage. The Cruskal-Wallis analysis was used to analyze the drug resistance rate of ATD strains of MTB within 6 years; the level of significance was set at *P* < 0.05.

## Results

### Demographic and clinical characteristics

The median age of 42 children with TBM was nine years, ranging from 5 months to 14 years.

Twenty-nine patients (69.0%) were between ages 5 to 14 years. The male: female ratio was 1:1. Thirty-four (81.0%) children belonged to ethnic minorities (Tibetan, Yi and Qiang), and none of the patients in this group received a BCG vaccination or had a BCG vaccination mark on the forearm. Only 2/42 (4.76%) of the patients who received BCG vaccination were Han Chinese from the main urban areas. Thirteen (31.0%) patients had a history of contact with an individual with pulmonary TB (Table 1).

**Table 1:** Demographic profile and clinical features of culture-confirmed children TBM in Southwest China, 2013–2018 (*n* = 42)

Variable	Total ***n*** = 42 (%)
Mean age; months (range)	93(5–168)
< 1 years	5(11.9)
1-5 years	8(19.0)
5-14 years	29(69.0)
Female	21(50.0)
BCG vaccination	2 (4.8)
Chinese Ethnic minorities	
Han	7(16.7)
Tibetan	27(64.3)
Yi	6(14.3)
Qiang	1(2.4)
History & Clinical Findings	
Temperature above 37.5 °C	38(90.5)
Headache	30(71.4)
Convulsions	4(9.5)
Disturbance of consciousness	14(33.3)
Cough	19(45.2)
Vomiting	22(52.4)
Weight loss	6(14.3)
Night sweats	3(7.1)
Neck stiffness	35(83.3)
CSF pressure > 200 mmH2O	30(71.4)
Recent close contact with an infectious TB case^a^	13(31.0)
Imaging	
Chest X-ray suggestive of TB	42(100.0)
Basal meningeal enhancement	12(28.6)
Cerebral oedema/Hydrocephalus	7(16.7)
Outcome	
Recovery	35(83.3)
Sequelae	6(14.3)
Death before hospital discharge	1(2.38)
Drug-induced Uric acid UA (> 430 μmol / L)	10(23.8)
ATD-induced hepatotoxicity^b^	6(14.3)

^a^ History of recent (within past year) close contact with an individual with pulmonary TB

^b^ The ATD induced hepatotoxicity is defined as ALT ≥3ULN

The most common symptoms of culture-confirmed children patients with TBM included fever (90.5%), headache (71.4%), neck stiffness (83.3%), vomiting (52.4%), cough (45.2%), disturbance of consciousness (33.3%) and varying degrees of convulsions, weight loss and night sweats (Table 1). Symptomatic resolution occurred in 35/42 (83.3%) patients following hospitalisation with an average length of stay (LOS) of 21 days. One patient (2.4%) died and 6/42 (14.3%) developed long term sequelae. During hospitalization, patients showed varying degrees of anti-tuberculosis drug-induced hyperuricemia (23.8%) and hepatotoxicity (14.3%) (Table 1).

### Laboratory and imaging findings

Chest X-rays changes were observed in all patients, and 18/42 (42.9%) patients had clinical evidence of extra-pulmonary and extracranial TB disease involving sites such as the cervical lymph nodes, pericardium, and peritoneum. Brain imaging showed varying degrees of basal meningeal enhancement (28.6%) and cerebral edema/Hydrocephalus (16.7%). In addition, thirty 30/42 (71.4%) patients had high CSF pressure >  200mmH2O (Table 1).

Laboratory testing revealed that 100.0, 88.1, 73.8 and 61.9% of patients had CSF total leucocyte count of > 20 cells/μL, CSF sugar levels < 2.2 mmol/L, proteins > 1.0 mg/dl and abnormal erythrocyte sedimentation rate (ESR), respectively. According to the results from 42 children patients with TBM, 17 (40.5%) had anemia, while Lactate dehydrogenase (54.8%), Hydroxybutyrate dehydrogenase (64.3%), C-Reactive protein (60.0%) and blood lactates acid (28.6%) were present to different degrees (Table 2).

**Table 2:** Laboratory findings of culture-confirmed children TBM in Southwest China, 2013–2018 (*n* = 42)

Variable	Total ***n*** = 42 (%)
**Cerebrospinal fluid results**	
Total leukocyte count cells / μl; median (range)	381(20–1300)
10 to 99	8(19.0)
100 to 399	19(45.2)
> 400	15(35.7)
Lymphocytes (cells× 106 / L) > 50%	32(76.2)
25 to 50	7(16.7)
51 to 75	17(40.5)
> 75	15(35.7)
Protein > 1.0 mg/dl	31(73.8)
Glucose < 2.2 mmol/L	37(88.1)
Chloride < 110 mmol/L	20(47.6)
**Blood results**	
ESR (Female > 20, male > 15 mm / hour)	26(61.9)
Lactate dehydrogenase (> 225 U/L)	23(54.8)
Hydroxybutyrate dehydrogenase (> 182 U/L)	27(64.3)
Anemia^a^	17(40.5)
C-Reactive protein (> 6 mg / L)	25(60.0)
Blood lactates acid (> 2.2 mmol/L)	12(28.6)

*ESR* erythrocyte sedimentation rate; ^a^0.5–4.99 yrs. Hemoglobin < 110 g /L, 5–11.99 yrs. Hemoglobin < 115 g/L, 12–14.99 yrs. Hemoglobin < 120 g/L [4]

### Drug resistance

CSF specimens from all 42 cases were culture-positive for *Mycobacterium tuberculosis*. DST was performed on all 42 culture positive specimens; resistance to any ATD was demonstrated in 13 / 42 (31%) isolates with 12 and 3 instances of first-line drug resistance and second-line drug resistance, respectively. In addition, two (4.8%) and 1 (2.4%) isolates were identified as MDR-TB and XDR-TB respectively, and the resistance to single ATD (from high to low) i.e., isoniazid (high-level resistance > 1.6 μg/ml) 10/42(23.8%), streptomycin (high-level resistance > 8.0 μg/ml) 4/42(9.5%), rifampicin (high-level resistance > 8.0 μg/ml) 3/42 (7.5%) and ofloxacin (high-level resistance > 2.0 μg/ml), amikacin (high-level resistance > 4.0 μg/ml), protionamid (high-level resistance > 40.0 μg/ml), moxifloxacin (high-level resistance > 2.0 μg/ml), rifabutin (high-level resistance > 3.0 μg/ml) were 1 (2.4%), respectively (Table 3). No changes were observed in the drug resistance rate of MTB strain against eleven ATDs in 6 years: isoniazid (*P* = 0.44), streptomycin (*P* = 0.29), rifampicin (*P* = 0.38), ofloxacin (P = 0.38), moxifloxacin (*P* = 0.14), protionamid (P = 0.38), rifabutin (P = 0.38), amikacin (P = 0.38). Because the drug resistance rate in capreomycin, ethambutol, and levofloxacin was 0 for 6 years, it was not statistically significant.

**Table 3:** Results of in vitro testing for drug-resistance for children TBM in Southwest China, 2013–2018 (*n* = 42)

Individual drug	No.(%) of isolates with resistant to (***n*** = 42)
Any drug resistance^a^	13 (31.0)
Any first-line drug resistance	12 (28.6)
Any second-line drug resistance	3 (7.1)
STR	4 (9.5)
INH	10 (23.8)
RIF	3 (7.1)
EMB	0 (0.0)
OFX	1 (2.4)
LFX	0 (0.0)
AMK	1 (2.4)
CM	0 (0.0)
PTO	1(2.4)
MFX	1 (2.4)
RFB	1 (2.4)
MDR (INH + RIF)	2 (4.8)
pre-XDR	0 (0.0)
XDR	1 (2.4)
INH + STR	2 (4.8)
INH + RIF + STR	1 (2.4)
INH + RIF + EMB	0 (0.0)
RIF + STR + EMB	0 (0.0)
INH + RIF + STR + EMB	0 (0.0)

*TBM* tuberculosis meningitis, *DST* drug sensitivity testing, *INH* isoniazid, *STR* streptomycin, *RIF* rifampicin, *EMB* ethambutol, *OFX* Ofloxacin, *LFX* Levofloxacin, *MFX* Moxifloxacin, *PTO* Protionamid, *RFB* Rifabutin, *AMK* Amikacin, *CM* Capreomycin, *MDR-TB* multidrug-resistant tuberculosis, *XDR* extensively drug-resistant tuberculosis; ^a^: Resistant to at least one drug

### Geographical distribution

**Fig. 1:**
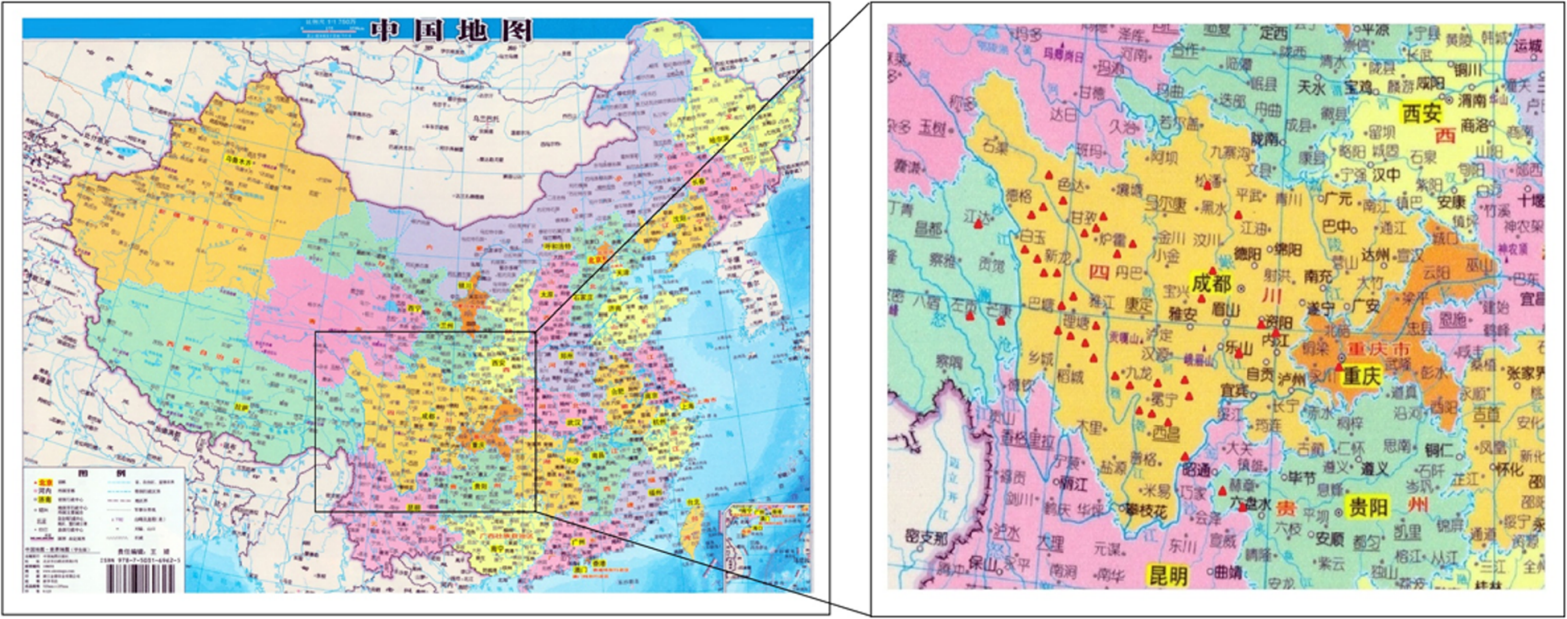
The geographical distribution of the People's Republic of China ( From map of China published by China map publishing house in 2014, which has been approved by the publishing and editing department ), the insert reports a magnification of the southwest of China where 42 study cases are present. Site locations (triangle) are red coloured according to the children TBM prevalence cases

The PHCCC is located in Chengdu city, the capital of Sichuan province. It is one of the oldest hospitals in the area and the authorized medical institution for treating TB in Chengdu city. The Geographical distribution figure revealed that most of the TBM cases in the current study were mainly from the Sichuan province, the southwest hinterland of the Chinese mainland. Meanwhile, most of the cases were mainly located in the ethnic areas of western Sichuan; there were also a few cases in the central and northern regions of Sichuan (Fig. 1).

## Discussion

China has the second-highest incidence of TB in the world. Located in the southwest hinterland of the Chinese mainland, Sichuan province is the gateway to the southwest of China. This area is a big multi-ethnic community with the second largest Tibetan region in China. Although the Chinese government has strengthened TB control in minority areas over recent years achieving modest success, infantile TBM remains a neglected field [5, 6]. WHO and numerous studies have reported that BCG vaccination can protect children from severe types of TB, such as TB meningitis and miliary TB [7, 8]. Administration of BCG vaccination is already implemented in most parts of China. It has been estimated that 76% of infants receive BCG vaccination at birth in China [9]. However, in the current study, more than 80% of children with TBM were from ethnic minority areas in the southwest of China, with no history of BCG vaccination or BCG vaccination marks on the forearm. Only 2/42 (4.76%) of the patients with a history of BCG vaccination were Han Chinese from the main urban areas. Due to the remote geographical location and difficulties with transportation, there are many residents of ethnic minorities within Sichuan province who are unable to readily access medical services. Consequently, there is poor awareness of disease prevention and many residents do not go to the hospital to give birth. All of these obstruct the government’s efforts to control TB, which is why BCG vaccination rates are low in these areas. Chinese government and WHO should also formulate corresponding TB control strategies for the population in these special areas.

Among forty-two children with a median age of 9 years, approximately 70% were between 5 and 14 years, and only 13 (31%) were under 5 years of age, which was somewhat different from some previous studies arguing that TBM mainly affects young children with the mean age ranging between 23 and 49 months [10–12]. This suggests that the BCG vaccine may have a certain protective effect not only on the incidence of TBM in early childhood but also in older children from different areas. It is possible that different regions, study population, and sample size may have a different age group structure.

In this study, there was no difference in distribution among male and female subjects. Among 42 patients, 1/42 (2.38%) patients died, while more than 83% patients successfully recovered during their stay at the hospital. There were no HIV-infected patients, while syphilis infection was found in one case. All the 42 cases had Chest X-rays suggestive of tuberculosis.

Following pulmonary infection with TB, children are more likely to develop disseminated disease and/or TBM due to their relatively lower immunity and development of initial non-specific symptoms that preclude early diagnosis. Fever (90.5%), headache (71.4%) and cough (45.2%) were the most common symptoms, while more severe symptoms like neck stiffness, vomiting, and disturbance of consciousness were present in 35/42 (83.3%), 22/42(52.4%), and 14/42 (33.3%), respectively. The occurrence of these symptoms was similar or higher to those observed in previous studies [13–16]. In addition, children with TBM during hospitalization showed varying degrees of anti-tuberculosis drug-induced hyperuricemia (23.8%) and hepatotoxicity (14.3%). These frequent adverse events associated with TB treatment ratio were higher in the current study compared to previous ones [6, 17].

Diagnosing TBM in children is difficult because of non-specific clinical features, insensitive laboratory tests and the low positive rate of CSF culture. Most of the reported cases of TBM lack the relevant bacteriological diagnostic basis [18–20], especially in children. In this study, the DST of TBM in children from southwest of China was high; ATD resistance and first-line drug resistance were 13 (31.0%) and 12 (28.6%) respectively, which was similar to adult tuberculous meningitis in Chengdu area. Besides, the drug resistance rate of isoniazid, streptomycin and rifampicin ranked among the first three [2]. This was different from the low rates of isoniazid resistance observed in children in some other parts of China [21, 22] and similar to Shaanxi province of China [23]. In our study, children with TBM and MDR-TB accounted for two (4.8) cases; while there was one case with XDR. This incidence is relatively lower compared with other regions of China, which may be due to regional differences and the different group of cases in our study [21–23]. No significant change was observed in the drug resistance rate of MTB strain against eleven ATDs over 6 years.

## Conclusions

We found that the children’s TBM in southwest of China was mainly concentrated in the minority areas of western Sichuan, and that the vaccination rate of BCG vaccine was very low, while the drug resistance rate was high. To the best of our knowledge, this is the first study that reported the drug-resistance patterns of children with TBM in southwest China, thus providing basis for the prevention and treatment of tuberculous meningitis. The government can use these results to further strengthen the prevention and control of TB in southwest China, especially in ethnic minority areas.

## Data Availability

The datasets used and/or analyzed during the current study are available from the corresponding author on reasonable request.

## Abbreviations

CSF: Cerebrospinal fluid
PHCCC: Public Health Clinical Center of Chengdu
NTM: Nontuberculous Mycobacteria
MTB: M.tuberculosis
DST: Drug sensitivity testing
BCG: Bacillus Calmette–Guérin
ATD: Anti-tuberculosis drug
MDR-TB: Multidrug-resistant tuberculosis
Pre-XDR: Pre-Extensively Drug Resistant
XDR: Extensively Drug Resistant
TB: Tuberculosis
INH: Isoniazid
RIF: Rifampicin
STR: Streptomycin
EMB: Ethambutol
OFX: Ofloxacin
LFX: Levofloxacin
MFX: Moxifloxacin
PTO: Prothionamide
RFB: Rifabutin
AMK: Amikacin
CM: Capreomycin
CLR: Clarithromycin
PNB: P-nitrobenzoic acid
TCH: Thiophene-2-carboxylic acid hydrazide
TB-DNA: Tuberculosis Deoxyribonucleic acid
EQA: External quality assessment
WHO: World Health Organization
